# Diagnostic value of four neuroendocrine markers in small cell neuroendocrine carcinomas of the cervix: a meta-analysis

**DOI:** 10.1038/s41598-020-72055-x

**Published:** 2020-09-11

**Authors:** Rui Huang, Li Yu, Chunying Zheng, Qingchun Liang, Suye Suye, Xue Yang, Huan Yin, Zhen Ren, Liye Shi, Zhibang Zhang, Hongliang Chen, Chun Fu

**Affiliations:** 1 Department of Obstetrics and Gynecology, Second Xiangya Hospital, Central South University, Changsha, Hunan China; 2 Department of Pathology, Second Xiangya Hospital, Central South University, Changsha, 410011 Hunan China

**Keywords:** Cancer, Biomarkers

## Abstract

Small cell neuroendocrine carcinoma of the cervix (SCNECC) is a highly invasive cervical cancer. The immunohistochemical criteria is an important aspect for assistant diagnosis of SCNECC. However, which markers can be appropriate selection for diagnosing SCNECC were not determined. The aim was to systematically evaluate expression levels of four neuroendocrine markers (containing synaptophysin (Syn), neural cell adhesion molecules (CD56), neuron-specific enolase (NSE) and chromograninA (CgA)) and to find out the appropriate selection for diagnosing SCNECC. Four English and three Chinese libraries were retrieved between 1984 and 2020. 23 studies about NSE, 36 studies about Syn, 23 studies about CD56 and 36 studies about CgA (all studies containing 581 patients) were eligible for meta-analyses. The pooled positive expression percentages (95% CI; I^2^) were as follows: 84.84% (79.41–90.27%; 76.7%) for Syn, 84.53% (79.43–89.96%; 37.5%) for CD56, 77.94% (69.13–86.76%; 83.5%) for NSE, and 72.90% (67.40–78.86%; 59.7%) for CgA. The positive proportions (95% CI; I^2^) ranked top three of simultaneous expressions of two markers were 87.75% (82.03–93.87%, 33.3%) for Syn and CD56, 70.92% (50.50–87.68%, 82.7%) for Syn and NSE, 65.65% (53.33–76.98%, 73.5%) for Syn and CgA. This confirms that Syn and CD56 are reliable indicators for diagnosing SCNECC.

## Introduction

Neuroendocrine carcinoma of the cervix (NECC) is an aggressive histological variant of cervical malignancy. Small cell NECC (SCNECC) is the most common and high–grade poorly differentiated histological subtype of NECC^1^. SCNECC is associated with adverse outcome in spite of even a small component in mixed carcinomas of the uterine cervix^2^. Therefore, accurate initial diagnosis of SCNECC is paramount. SCNECC has its unique growth characteristic that the cancer cells have the capacity to invade the stroma extensively even in the early stage^1,3^. This may result in negative cytology and increase the difficulty of clinical diagnosis. Pathomorphological diagnosis is the basis for SCNECC, the immunohistochemical (IHC) criteria is an important aspect for the diagnosis too^3^.

To establish the SCNECC diagnosis, at least one or two positive staining neuroendocrine markers is recommended^1,4–6^. IHC staining for neuroendocrine markers include synaptophysin (Syn), neural cell adhesion molecules (CD56), neuron-specific enolase (NSE) and chromograninA (CgA), positive expression of which indicates the neoplasms arising from cells of the neuroendocrine system^7,8^. The above four neuroendocrine markers have been widely used for the assistant diagnosis of SCNECC. However, due to the low incidence of SCNECC, the relative literatures were clinical case reports or case series^1,3^. In addition, there are differences in values of positive expression rate among individual studies. Accordingly, the true levels of the four neuroendocrine markers may not be accurate by direct quantitative assessment of each study. It is also difficult to select the appropriate neuroendocrine markers to assist to diagnose SCNECC. Hence we performed a meta–analysis to evaluate the IHC expression of Syn, CD56, NSE and CgA, and aimed to provide an appropriate selection of neuroendocrine markers for assistant diagnosis of SCNECC.

## Methods

### Literature search

**Figure 1:**
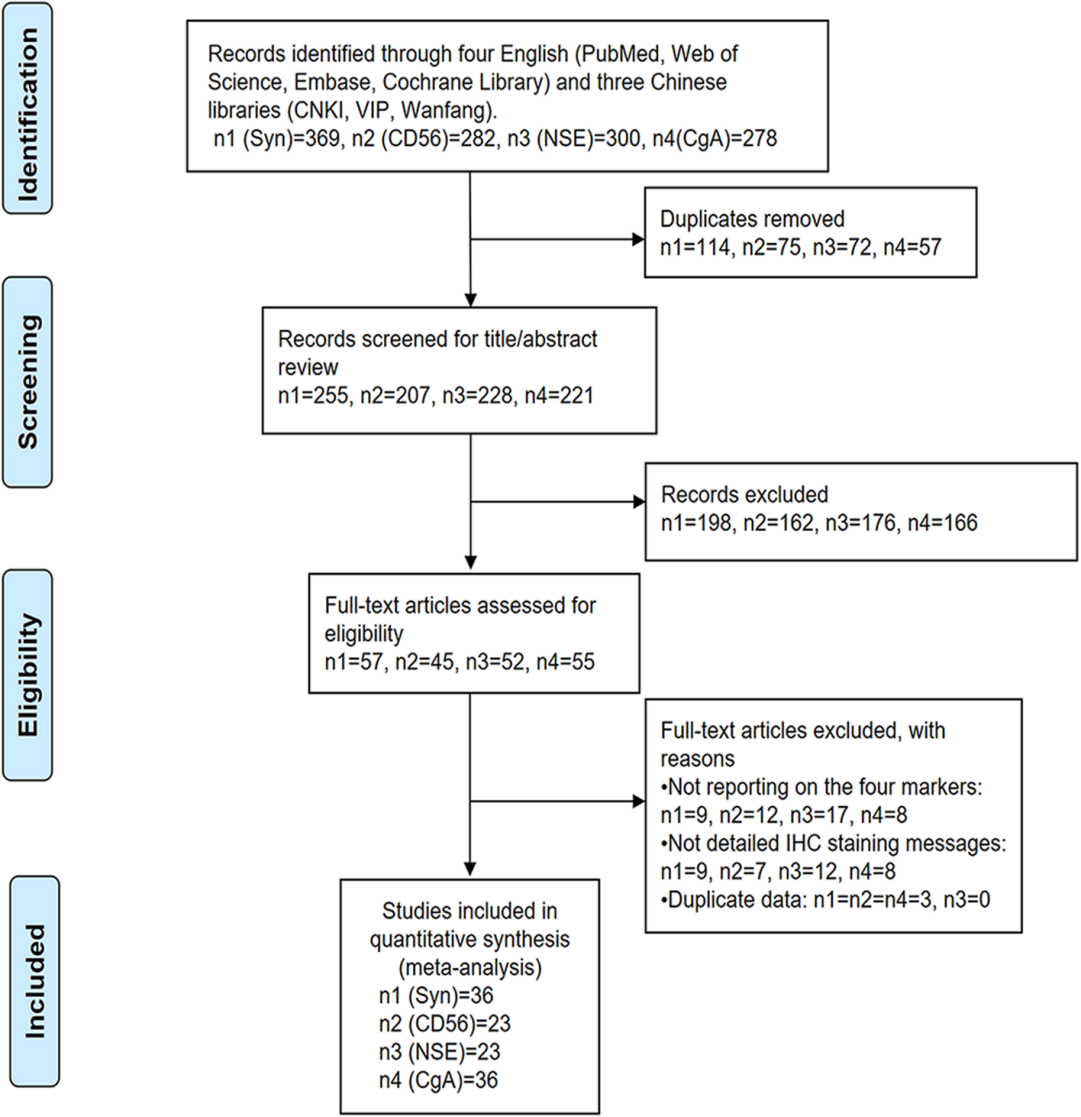
The screening flow chart of literature about small cell neuroendocrine carcinoma of the cervix (SCNECC). n1, n2, n3 and n4 respectively represent the number of the literature about Syn, CD56, NSE and CgA in each screening step. *CNKI* China National Knowledge Infrastructure; *SCNECC* small cell neuroendocrine carcinoma of the cervix; *Syn* synaptophysin; *CD56* neural cell adhesion molecules; *NSE* neuron-specific enolase; *CgA* chromograninA; *IHC* immunohistochemical staining.

The study was approved by the Ethics Committee of Second Xiangya hospital. We only retrospectively extracted the clinical and pathological data of patients, which had no impact on the outcome of patients. The study was conducted according to the Preferred Reporting Items for Systematic Reviews and Meta-analyses (PRISMA) guidelines. Patients and public were not involved in this study. We interrogated four English libraries (the PubMed, Cochrane library, Web of science, and EMbase databases) and three Chinese libraries (the China National Knowledge Infrastructure (CNKI), VIP, and Wanfang databases) to perform a comprehensive search from inception to 20 January 2020. The broad search strategy used combinations of the following key terms: “Cervical tumor”, “small cell carcinoma”, “neuroendocrine carcinoma”, “neuroendocrine marker”. In order to interpret the results more objectively, the titles and abstracts were carefully screened according to the screening flow chart showed in the Fig. 1.

### Eligibility criteria

The inclusion criteria were as follows: (i) English or Chinese published clinical studies; (ii) SCNECC patients confirmed by pathological diagnosis without age or racial restrictions; (iii) detailed IHC information with the positive expression rate of at least one of four neuroendocrine markers (Syn, CD56, NSE and CgA). The exclusion criteria were as follows: (i) case report of individual patient; (ii) systematic review or duplicate data; (iii) no detailed messages of IHC staining; (iv) non-SCNECC patients; (v) literature on basic research and animal studies. The research was still included if the study group was SCNECC patients and the control group was non-SCNECC patients.

All potentially relevant abstracts or full articles were reviewed independently by two researchers. When discrepancies between researchers occurred for inclusion or exclusion, discussion was conducted and disagreements were resolved by consensus. Quality assessment of all qualified literature was then done.

### Data extraction

Information including types of studies, name of first author, institution, publication year and clinical data of patients was analyzed in each study.The clinical data were consisted of age, tumor stage, histopathological type, IHC results, treatment and prognosis.

We searched the definition of IHC positive staining in each study and found three methods of description. The first method only mentioned the positive expression without specific description. The second defined positive staining by positive staining percentage of tumor cells. When the staining rate of neuroendocrine markers was more than 5% or 10%, the expression of neuroendocrine markers was positive. The third was definition of positive staining by a four-point scale. Staining was graded as 0, 1 + (less than 5% or 10% tumor cells), 2 + (5% or 10–50% tumor cells), 3 + (more than 50% tumor cells) respectively. The positive expression of neuroendocrine markers by any of the above three methods was regarded as positive expression.

The positive expression rate of individual neuroendocrine marker in one study was defined as follows: number of positive expression SCNECC cases/number of tested SCNECC cases (percentage). In addition, there are 6 combinations of the two markers, which are Syn and CD56, Syn and NSE, Syn and CgA, CD56 and NSE, CD56 and CgA, NSE and CgA. Similarly, the positive expression rate of two markers can be calculated as followed: number of simultaneous positive expression cases/number of tested cases in one study (percentage). If the study included patients with non-SCNECC, we tried to calculate the sensitivity and specificity of neuroendocrine markers expression.

### Statistical analysis

All raw data extracted from the predetermined studies were managed using MetaProp function in statistical software R 3.5.0. The detailed approach of meta-analysis was shown in Supplementary Fig. 1. The first step was to determine if a transformation of raw rate is needed. The converted rate was calculated with optimum one of the four proportion transform methods (log, logit, arcsine, and dsrsine) if the raw rate does not satisfy the normal distribution. Then the pooled expression proportions with 95% confidence intervals (CIs) of one marker or two markers could be determined using the appropriate transformation to give the effective value. A random effect model was selected when *p* ≤ 0.05 and a fixed effect model was used when *p* > 0.05.

Additionally, we also measured the effect of heterogeneity between the included studies using I^2^ = 100% × (Q − df)/Q. I^2^ value of 25%, 50%, and 75% were considered as low, moderate, and high degrees of heterogeneity respectively. The results of meta-analysis were presented in forest maps. Funnel plot asymmetry was assessed by the Egger's linear regression test. The results provided publication bias results for this meta-analysis. *p* < 0.05 was considered significant bias.

## Results

### Study searches and characteristics

A total of 369 literatures about Syn, 282 literatures about CD56, 300 literatures about NSE, and 278 literatures about CgA were identified as potentially eligible for inclusion. A flow diagram of the study selection is shown in Fig. 1. Of the remaining 42 studies warranting furthering review, 41 studies were case series and 1 study was a case report. 42 studies were harmonized for inclusion criteria, comprising 29 from Asia (China, Japan, Indian, and Thailand), 4 from Europe (England, Germany, Poland), 8 from the United States of America and 1 from Canada. All included studies were retrospective and a total of 581 patients were enrolled in the stratified meta-analyses. Characteristics of studies used in the research are enumerated in the Table 1.

**Table 1:** The general and clinical characteristics of studies about small cell neuroendocrine carcinomas of the cervix (SCNECC).

First author and year	Country	Institution	Case number	Age (y)	Histological type	FIGO stage	Overall survival (m), death(n)
Chen 1994	China	National Taiwan University Hospital	6	U	SCNECC	U	U, U
Cheng 2008	China	Peking Union Medical College	7	24–61	SCNECC	1 IB1, 2 IB2, 2 IIB, 2 IIIB	5–64, 3
Conner 2002	USA	The University of Alabama at Birmingham	23	23–63	SCNECC	IB-IIB	6–273, 15
Deng 2010	China	Shanghai First Maternity and Infant Hospital	9	31–54	SCNECC	6 IBl, 2 IB2, 1 IVB	6–104, 1
Emerson 2015	USA	Indiana University School of Medicine	11	18–79	5SCNECC 6Mixed SCNECC	U	9–122, 5
Fujii 1986	Japan	Kyoto University	2	30, 31	1SCNECC 1Mixed SCNECC	2 IB	6–14, 2
Ganesan 2016	UK	Birmingham Women’s NHS Foundation Trust	23	23–79	16SCNECC 7Mixed SCNECC	22 I-II + , 1 U	0.2–57.3, U
Giorgadze 2012	USA	Wayne State University	3	34–54	SCNECC	1 IIIB, 2 U	U, 1
Horn 2006	Germany	University of Leipzig	9	24–61	4SCNECC 5Mixed SCNECC	6 IB1, 1 IB2, 1 IIA, 1 IIB	15.6–151.2, 4
Inoue 1985	Japan	Osaka University Medical School	6	U	SCNECC	IA-IIIB	U, U
Ishida 2004	Japan	Yamagata University School of Medicine	10	30–59	3SCNECC 7Mixed SCNECC	U	U, U
Kajiwara 2008	Japan	Saitama Medical University International Medical Center	5	23–73	3SCNECC 2Mixed SCNECC	1 IB1, 1 IIA, 1 IIB, 1 IIIA, 1 IIIB	11–18, 3
Kuji 2017	Japan	Shizuoka Cancer Center Hospital	29	26–76	24SCNECC 5Mixed SCNECC	IB1-IVB	U, U
Lenczewski 2001	Poland	Medical Academy of Białystok	6	U	SCNECC	IB-IIA	U, U
Li 2011	China	Sun Yat-Sen University	25	24–65	19SCNECC 6Mixed SCNECC	12 IB1, 5 IB2, 5 IIA, 3 IIB	5–62, U
Li 2013	China	Shanghai First Maternity and Infant Hospital	6	31–74	5SCNECC 1Mixed SCNECC	4 IB1, 1 IIA, 1 IIB	U, U
Qin 2011	China	Tumor Hospital of Guangxi Medical University	12	28–57	SCNECC	2 IB1, 3 IB2, 1 IIA, 1 IIB, 3 IIIB, 2 IV	0.6–21.8, 4
Rekhi 2012	India	Tata Memorial Hospital	25	23–69	20SCNECC 5Mixed SCNECC	13 U, 2 IB1, 1 IIA, 3 IIB, 2 IIIA, 1 IIIB, 2 IVA, 1 IVB	3–36, 4
Sato 2003	Japan	Miyazaki Prefectural Hospital	2	47, 42	SCNECC	1 IB, 1 IIA	U, 1
Sheridan 1996	UK	Weston Park Hospital	5	29–41	SCNECC	U	U, U
Sitthinamsuwan 2013	Thailand	Medicine Siriraj Hospital	11	34–55	8SCNECC 3Mixed SCNECC	4 IB, 5 IIB, 1 IIIB, 1 IVB	4–60, 7
Stoler 1991	USA	The University of Rochester	20	U	SCNECC	U	U, U
Straughn 2001	USA	University of Alabama	16	23–53	SCNECC	11 IB, 4 IIB, 1 IV	6–264, 11
Tsunoda 2005	Japan	School of Medicine, Kitasato University	11	32–65	SCNECC	4 IB, 3 IIB, 3 IIIB,1 IV	4–144, 8
Ueda 1989	Japan	Osaka University Medical School	10	U	SCNECC	U	U, U
Van 1988	USA	University of Kentucky Medical Center	15	U	SCNECC	IB-IVB	U, U
Viswanathan 2004	USA	Brigham and Women’s Hospital	21	26–78	SCNECC	10 IB1, 5 IB2, 2IIA, 1 IIB, 3 IIIB	6–209, U
Wang 2013	China	Sichuan Cancer Hospital	13	21–62	SCNECC	U	U, U
Xing 2018	USA	The University of Alabama	10	28–68	SCNECC	U	2–126, 4
Hu 2018	China	Zhengzhou University	35	29–76	28SCNECC 7Mixed SCNECC	6 IB1, 8 IB2, 12 IIA, 4 IIB, 3 IIIA, 1 IVA, 1 IVB	U, 8
Han 2018	China	Beijing Obstetrics and Gynecology Hospital	18	24–66	SCNECC	6 IB1, 5 IB2, 4 IIA2, 2 IIB,1 IIIB	U, 8
Yang 2018	China	Guigang City People’ Hospital	18	25–74	17SCNECC 1Mixed SCNECC	5 IB, 6 IIA, 3 IIB, 1 IIIA, 1 IIIB, 2 IVB	U, 4
Zeng 2018	China	Guizhou Medical University	8	28–51	6SCNECC 2Mixed SCNECC	4 IB1, 1 IB2, 1IIA2, 2 IIB	U, 4
Zhi 2018	China	Department of Pathology of Xi’an No. Hospital	10	31–54	SCNECC	7 IB1, 1 IB2, 2 IIA	U, 4
Wang 2019	China	Nanjing Medical University	26	45(median)	SCNECC	9 IA-IIA, 17 IIB-IV	U, 16
Tong 2018	China	Guizhou Provincial People’ Hospital	6	29–56	SCNECC	1 IB1, 1 IB2, 2 IIB, 1 IVB, 1 U	U, 1
Wang 2018	China	Chaohu Hospital of Anhui Medical University	18	31–55	SCNECC	4 IB1, 7 IB2, 2 IIA1, 3 IIA2, 2 IVA	U, U
Li 2015	China	Sun Yat-sen University	26	31–67	12SCNECC 14Mixed SCNECC	10 IB1,8 IB2, 2 IIA2, 4 IIIB, 2 IVB	3–42, 5
Morgan 2019	Canada	Department of Laboratory Medicine and Pathobiology	10	25–80	SCNECC	U	U, U
Liu 2018	China	General Hospital of Jinan Military Command	23	31–74	12SCNECC, 11Mixed SCNECC	U	U, U
Li 2018	China	Peking Union Medical College Hospital	26	U	SCNECC	I-IIA	U, U
Jain 2019	India	Nepal Cancer Hospital and Research Center	6	28–67	SCNECC	U	U, U

*Note* Mixed SCNECC included one or more components besides small cell neuroendocrine carcinoma, such as large cell neuroendocrine carcinoma, adenocarcinoa, squamous carcinoma, adenosquamous carcinoma.

*U* unknown; *OS* overall survival; *m* month; *y* year; *n* number; *UK* United Kingdom; *USA* United States of America; *FIGO* International Federation of Gynecology and Obstetrics.

For the 42 studies in the meta-analysis, 36 SCNECC studies involved Syn^1–23,30–42^, 23 studies involved CD56^1,2,6,7,9,10,12,13,15,17–19,30,32–38,40–42^, 23 studies involved NSE^1–3,6,7,12,13,15,17,21,22,24–30,32–34,38,42^and 36 studies involved CgA^1–7,9,10,12,13,15–23,25,28–42^. 26 studies provided information about positive expression rates for two markers. There were 15 studies about Syn and CD56^1,6,7,9,12,13,15,17–19,32–34,36,42^, 15 studies about Syn and NSE^1,3,6,7,12,13,17,18,22,25,32–34,38,42^, 23 studies about Syn and CgA^1,3,4,6,7,9,12,13,15,17–20,22,23,25,31–34,36,39,42^, 9 studies about CD56 and NSE^1,6,7,12,17,18,33,38,42^, 12 studies about CD56 and CgA^1,6,7,9,12,13,15,17–19,33,42^and 15 studies about NSE and CgA^1,3,6,7,12,13,17,18,21,22,25,29,33,38,42^. The positive expressions of one marker and two markers are illustrated in the Table 2. For the description of IHC positive staining, 30 studies took the first method, 2 studies the second method, and 10 studies the third method respectively.

**Table 2:** The positive expressions of neuroendocrine markers in SCNECC.

First author and year	N1/N2 (%)				N3/N4 (%)					
	Syn (N = 36)	CD56 (N = 23)	NSE (N = 23)	CgA (N = 36)	Syn + CD56 (N = 15)	Syn + NSE (N = 15)	Syn + CgA (N = 23)	CD56 + NSE (N = 9)	CD56 + CgA (N = 12)	NSE + CgA (N = 15)
Chen 1994	–	–	4/6 (66.67)	–	–	–	–	–	–	–
Cheng 2008	4/7 (57.14)	–	7/7 (100.00)	2/7 (28.57)	–	5/5 (100.00)	2/2 (100.00)	–	–	2/2 (100.00)
Conner 2002	13/23 (56.52)	–	–	10/23 (43.48)	–	–	–	–	–	–
Deng 2010	9/9 (100.00)	9/9 (100.00)	9/9 (100.00)	4/9 (44.44)	9/9 (100.00)	9/9 (100.00)	4/9 (44.44)	9/9 (100.00)	4/9 (44.44)	4/9 (44.44)
Emerson 2015	9/11 (81.82)	–	–	6/11 (54.55)	–	–	6/11 (54.55)	–	–	–
Fujii 1986	–	–	0/2 (0.00)	–	–	–	–	–	–	–
Ganesan 2016	19/23 (82.61)	15/23 (65.22)	–	14/23 (60.87)	–	–	–	–	–	–
Giorgadze 2012	3/3 (100.00)	1/2 (50.00)	2/2 (100.00)	2/3 (66.67)	1/2 (50.00)	2/2 (100.00)	2/3 (66.67)	0/1 (0.00)	1/2 (50.00)	1/2 (50.00)
Horn 2006	7/9 (77.78)	8/9 (88.89)	7/9 (77.78)	7/9 (77.78)	–	–	–	–	–	–
Inoue 1985	–	–	6/6 (100.00)	–	–	–	–	–	–	–
Ishida 2004	7/10 (70.00)	–	–	9/10 (90.00)	–	–	6/10 (60.00)	–	–	–
Kajiwara 2008	5/5 (100.00)	4/5 (80.00)	–	4/5 (80.00)	4/5 (80.00)	–	4/5 (80.00)	–	3/5 (60.00)	–
Kuji 2017	25/29 (86.21)	23/29 (79.31)	–	25/29 (86.21)	–	–	–	–	–	–
Lenczewski 2001	6/6 (100.00)	–	–	–	–	–	–	–	–	–
Li 2011	24/25 (96.00)	17/25 (68.00)	25/25(100.00)	19/25 (76.00)	17/25 (68.00)	24/25 (96.00)	19/25 (76.00)	17/25 (68.00)	14/25 (56.00)	19/25 (76.00)
Li 2013	6/6 (100.00)	5/6 (83.33)	–	5/6 (83.33)	5/6 (83.33)	–	5/6 (83.33)	–	4/6 (66.67)	–
Qin 2011	9/10 (90.00)	4/4 (100.00)	4/4 (100.00)	8/12 (66.67)	2/2 (100.00)	2/3 (66.67)	6/10 (60.00)	1/1 (100.00)	1/4 (25.00)	4/4 (100)
Rekhi 2012	14/25 (56.00)	6/6 (100.00)	5/5 (100.00)	15/24 (62.50)	4/6 (66.67)	2/4 (50.0)	11/23 (47.83)	–	4/6 (66.67)	2/4 (50.00)
Sato 2003	1/2 (50.00)	–	–	–	–	–	–	–	–	–
Sheridan 1996	–	–	2/5 (40.00)	3/5 (60.00)	–	0/5 (0.00)	0/5 (0.00)	–	–	2/5 (40.00)
Sitthinamsuwan 2013	8/11 (72.73)	8/11 (72.73)	9/11 (81.82)	7/11 (63.64)	6/11 (54.55)	6/11 (54.55)	6/11 (54.55)	6/11 (54.55)	4/11 (36.36)	5/11 (45.45)
Stoler 1991	5/20 (25.00)	–	18/20 (90.00)	13/20 (65.00)	–	–	–	–	–	8/20 (40.00)
Straughn 2001	8/16 (50.00)	–	12/16 (75.00)	8/16 (50.00)	–	6/13 (46.15)	4/13 (30.77)	–	–	7/13 (53.85)
Tsunoda 2005	8/11 (72.73)	6/11 (54.55)	9/11 (81.82)	7/11 (63.64)	6/11 (54.55)	6/11 (54.55)	5/11 (45.45)	4/11 (36.36)	3/11 (27.27)	6/11 (54.55)
Ueda 1989	–	–	9/10 (90.00)	4/10 (40.00)	–	–	–	–	–	4/10 (40.00)
Van 1988	–	–	5/15 (33.33)	3/15 (20.00)	–	–	–	–	–	–
Viswanathan 2004	19/21 (90.48)	15/21 (71.43)	–	16/21 (76.19)	14/21 (66.67)	–	15/21 (71.43)	–	11/21 (52.38)	–
Wang 2013	13/13(100.00)	–	–	9/13 (69.23)	–	–	–	–	–	–
Xing 2018	10/10(100.00)	–	–	8/10 (80.00)	–	–	8/10 (80.00)	–	–	–
Hu 2018	28/35 (80.00)	27/35 (77.14)	30/35 (85.71)	20/35 (57.14)	–	–	–	–	–	–
Han 2018	18/18(100.00)	–	–	18/18(100.00)	–	–	18/18(100.00)	–	–	–
Yang 2018	18/18(100.00)	17/18 (94.44)	9/16 (56.25)	10/16 (62.50)	17/18 (94.44)	9/16 (56.25)	9/16 (56.25)	–	–	–
Zeng 2018	7/8 (87.50)	5/7 (71.43)	0/2 (0.00)	5/8 (62.50)	4/7 (57.14)	0/2 (0.00)	4/8 (50.00)	0/2 (0.00)	3/7 (42.86)	0/2 (0.00)
Zhi 2018	10/10(100.00)	6/7 (85.71)	5/8 (62.50)	9/10 (90.00)	6/7 (85.71)	5/8 (62.50)	9/10 (90.00)	–	–	–
Wang 2019	19/26 (73.08)	20/26 (76.92)	–	18/26 (69.23)	–	–	–	–	–	–
Tong 2018	6/6 (100.00)	5/6 (83.33)	–	3/6 (50.00)	5/6 (83.33)	–	3/6 (50.00)	–	–	–
Wang 2018	9/18 (50.00)	9/18 (50.00)	–	12/18 (66.67)	–	–	–	–	–	–
Li 2015	24/26 (92.31)	24/26 (92.31)	26/26(100.00)	18/26 (69.23)	–	24/26 (92.31)	–	24/26 (92.31)	–	18/26 (69.23)
Morgan 2019	10/10(100.00)	–	–	5/8 (62.50)	–	–	5/8 (62.50)	–	–	–
Liu 2018	21/23 (91.30)	21/23 (91.30)	–	19/23 (82.61)	–	–	–	–	–	–
Li 2018	19/26 (73.08)	12/21 (57.14)	–	19/24 (79.17)	–	–	–	–	–	–
Jain 2019	6/6 (100.00)	4/4 (100.00)	3/3 (100.00)	6/6 (100.00)	4/4 (100.00)	3/3 (100.00)	6/6 (100.00)	1/1 (100.00)	4/4 (100.00)	3/3 (100.00)

*Note* N2 represnts the number of cases stained by Syn or CD56 or NSE or CgA in each study, and N1 represnts the positive staining ones; N4 represents the number of case stained by a combination of two neuroendocrine markers (Syn + CD56, Syn + NSE, Syn + CgA, CD56 + NSE, CD56 + CgA, NSE + CgA), and N3 represnts the positive staining cases correspondingly.

N represents the number of studies included for meta analyses of one marker (Syn or CD56 or NSE or CgA) or each combination of two markers (Syn + CD56, Syn + NSE, Syn + CgA,

CD56 + NSE, CD56 + CgA, NSE + CgA).

### Meta–analyses of expression percentage of neuroendocrine markers

**Figure 2:**
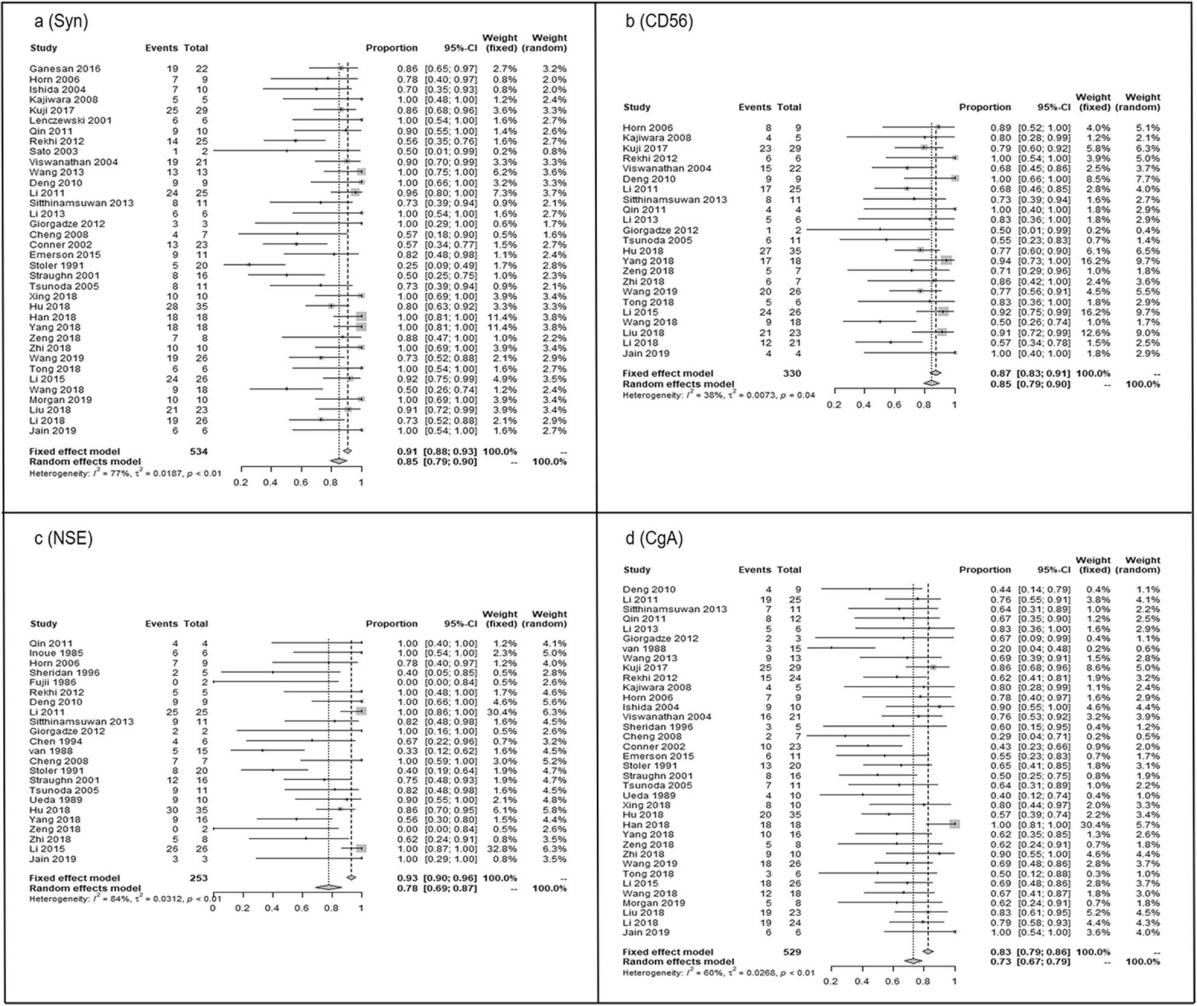
Forest plots of expression proportion (95% CI) for single neuroendocrine markers in small cell neuroendocrine carcinoma of the cervix (SCNECC). (**a**) (Syn), (**b**) (CD56), (**c**) (NSE) and (**d**) (CgA).The weight represents the percentage of the results of a single study in the overall results. Generally, the larger the number of cases in a single study, the greater its weight in the overall population. *CI* confidence interval; *Syn* synaptophysin; *CD56* neural cell adhesion molecules; *NSE* neuron-specific enolase; *CgA* chromograninA.

**Figure 3:**
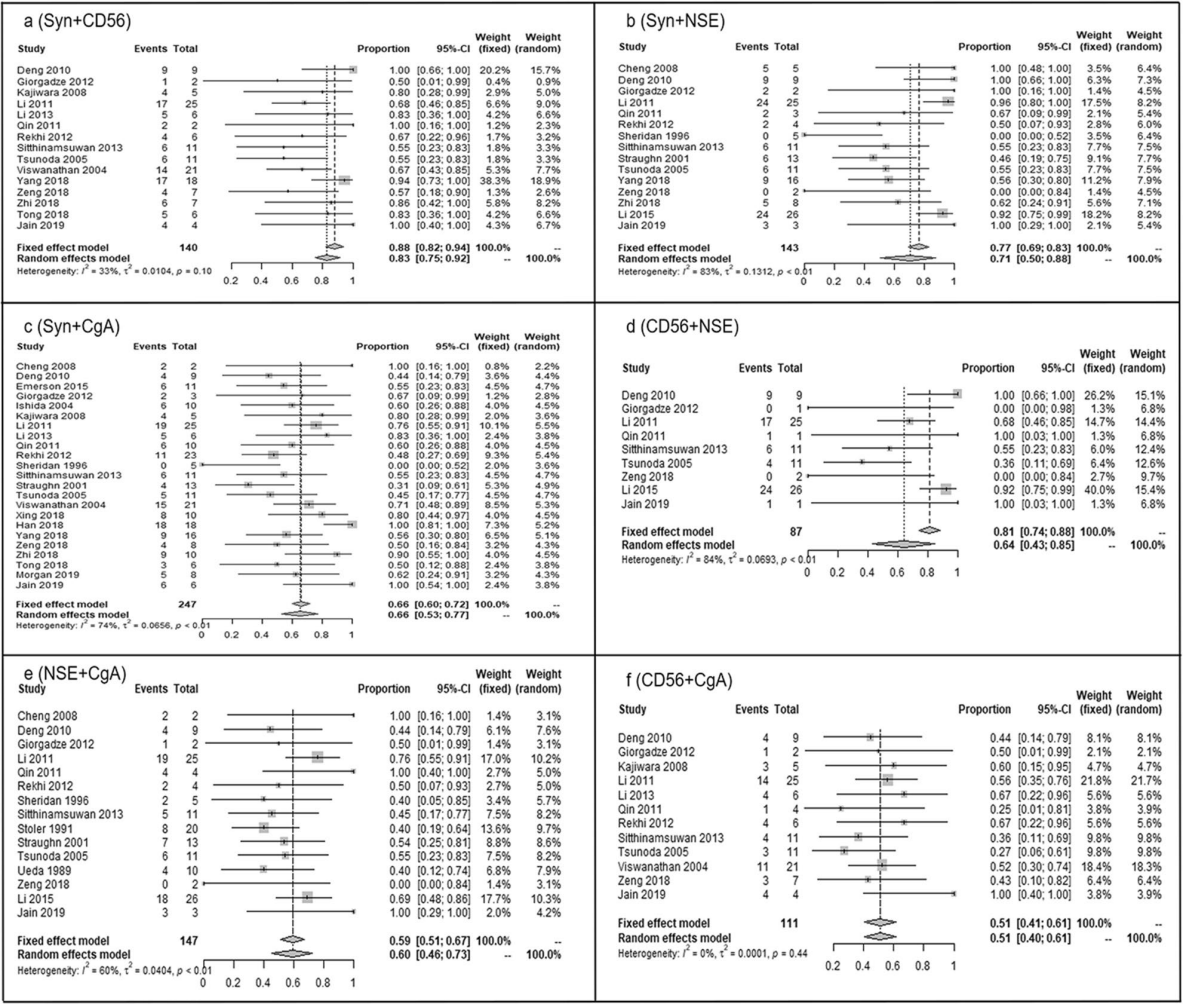
Forest plots of expression proportion (95% CI) for combination of two neuroendocrine markers in small cell neuroendocrine carcinoma of the cervix (SCNECC). (**a**) (Syn + CD56), (**b**) (Syn + NSE), (**c**) (Syn + CgA), (**d**) (CD56 + NSE), (**e**) (NSE + CgA), (**f**) (CD56 + CgA). The weight represents the percentage of the results of a single study in the overall results. Generally, the larger the number of cases in a single study, the greater its weight in the overall population. *CI* confidence interval; *Syn* synaptophysin; *CD56* neural cell adhesion molecules; *NSE* neuron-specific enolase; *CgA* chromograninA.

Results of the pooled positive expression percentages (95% CI) were as follows: 84.84% (79.41% to 90.27%) for Syn, 84.53% (79.43% to 89.96%) for CD56, 77.94% (69.13% to 86.76%) for NSE, and 72.90% (67.40% to 78.86%) for CgA (Fig. 2a–d). The transform methods of six combination expression levels were showed separately in Table 3. The positive proportions with 95% CIs expressed by the two markers from high to low were 87.75% (82.03% to 93.87%) for Syn and CD56, 70.92% (50.50% to 87.68%) for Syn and NSE, 65.65% (53.33% to 76.98%) for Syn and CgA, 64.09% (43.38% to 84.79%) for CD56 and NSE, 59.55% (45.53% to 72.81%) for NSE and CgA, 50.98% (40.52% to 61.39%) for CD56 and CgA (Fig. 3a–f).

**Table 3:** Meta-analyses results of neuroendocrine markers in small cell neuroendocrine caricinoma of the cervix.

Markers	Transformation methods	P value	I^2^ value (%)	Model	Pooled expression proportions with 95% CI	P value of dissymmetry test
Syn	Raw rate	< 0.0001	76.7	Random effect	0.8484 (0.7941–0.9027)	0.0002
CD56	Log	0.0367	37.5	Random effect	0.8453 (0.7943–0.8996)	0.0018
NSE	Raw rate	< 0.0001	83.5	Random effect	0.7794 (0.6913–0.8676)	0.0003
CgA	Log	< 0.0001	59.7	Random effect	0.7290 (0.6740–0.7886)	< 0.0001
Syn + CD56	Log	0.1020	33.3	Fixed effect	0.8775 (0.8203–0.9387)	0.0047
Syn + NSE	Arcsine	< 0.0001	82.7	Random effect	0.7092 (0.5050–0.8768)	0.3252
Syn + CgA	Arcsine	< 0.0001	73.5	Random effect	0.6565 (0.5333–0.7698)	0.8454
CD56 + NSE	Raw rate	< 0.0001	83.7	Random effect	0.6409 (0.4338–0.8479)	N
NSE + CgA	Arcsine	0.0014	60.2	Random effect	0.5955 (0.4553–0.7281)	0.8831
CD56 + CgA	Darcsin	0.4393	0.4	Fixed effect	0.5098 (0.4052–0.6139)	0.6312

*Syn* synaptophysin; *CD56* neural cell adhesion molecules; *NSE* neuron-specific enolase; *CgA* chromograninA; *CI* confidence interval; *N* null because the sample number is too small.

### Test of heterogeneity

The heterogeneity among studies included for the meta-analysis of Syn was evaluated firstly. As seen in Table 3, a random effect model was used because I^2^ was 76.7% with *p* value < 0.05. Similarly, random effect models were selected for other three single markers and four combinations (Syn and NSE, Syn and CgA, CD56 and NSE, NSE and CgA). While, fixed effect models were selected for two combinations (Syn and CD56, CD56 and CgA) since *p* value > 0.05.

### The sensitivity and specificity of neuroendocrine markers expression

Only 6 studies described IHC staining of neuroendocrinenon markers in non-SCNECC patients^8–10,24,28,29^. In three of these studies, non-SCNECC patients were larger cell NECC (LCNECC) patients^8–10^. So the sensitivity and specificity of neuroendocrine markers can be calculated simultaneously in only 3 studies. The results were shown in Supplementary Table 1. This part of results was not further meta-analyzed because of the small sample size.

### Quality assessment

42 studies included 39 full articles and 3 abstracts. Only the full articles were performed for quality assessment by appraisal checklists^43^, which included two different assessment forms, separately used for case series (38 studies) and case reports (1 study). The quality assessment of case series was shown in Supplementary Table 2. 80% enrolled studies gave positive response to 7 questions, and all studies satisfied 3 questions among them. The quality assessment of one case report was shown in Supplementary Table 3. The results showed that only the adverse events or unanticipated events were not identified in the study (question 7).

### Publication bias

**Figure 4:**
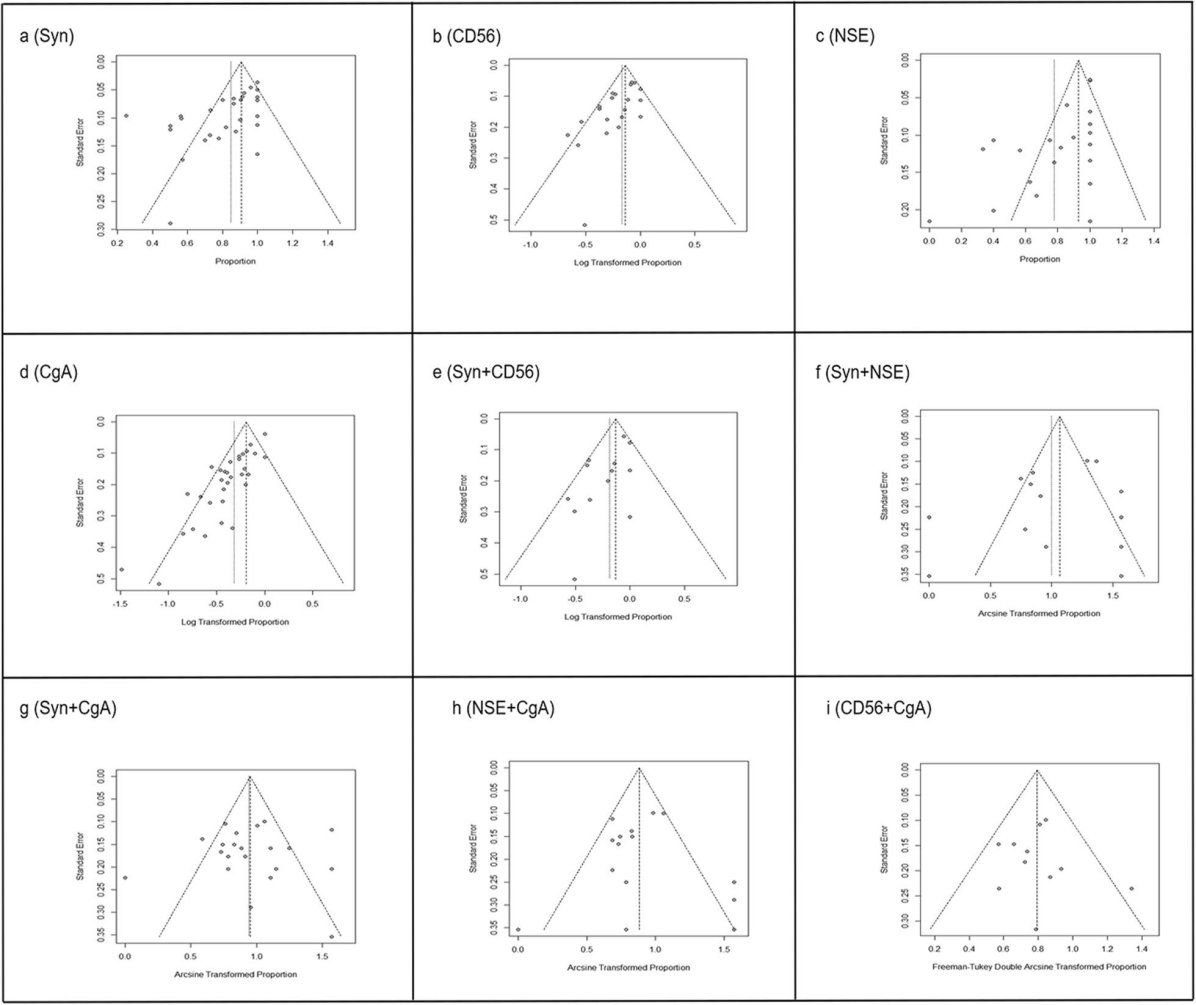
Funnel plots estimating possible publication bias. The sample size of the combination of CD56 + NSE is too small to evaluate the publication bias. One requirement of asymmetric analysis is that the case number of included studies is greater than nine. So we eliminated the analysis of studies of CD56 and NSE for whose sample size did not meet analytical criteria.

Publication bias was evaluated via Egger’s test. The Egger’s test (*p* > 0.05) suggested no significant publication bias. The results showed that the most of the literatures related to combined groups had no significant publication bias (Table 3, Fig. 4e–i). While, literatures about single markers had significant publication bias (Table 3, Fig. 4a-d).

## Discussion

**Figure 5:**
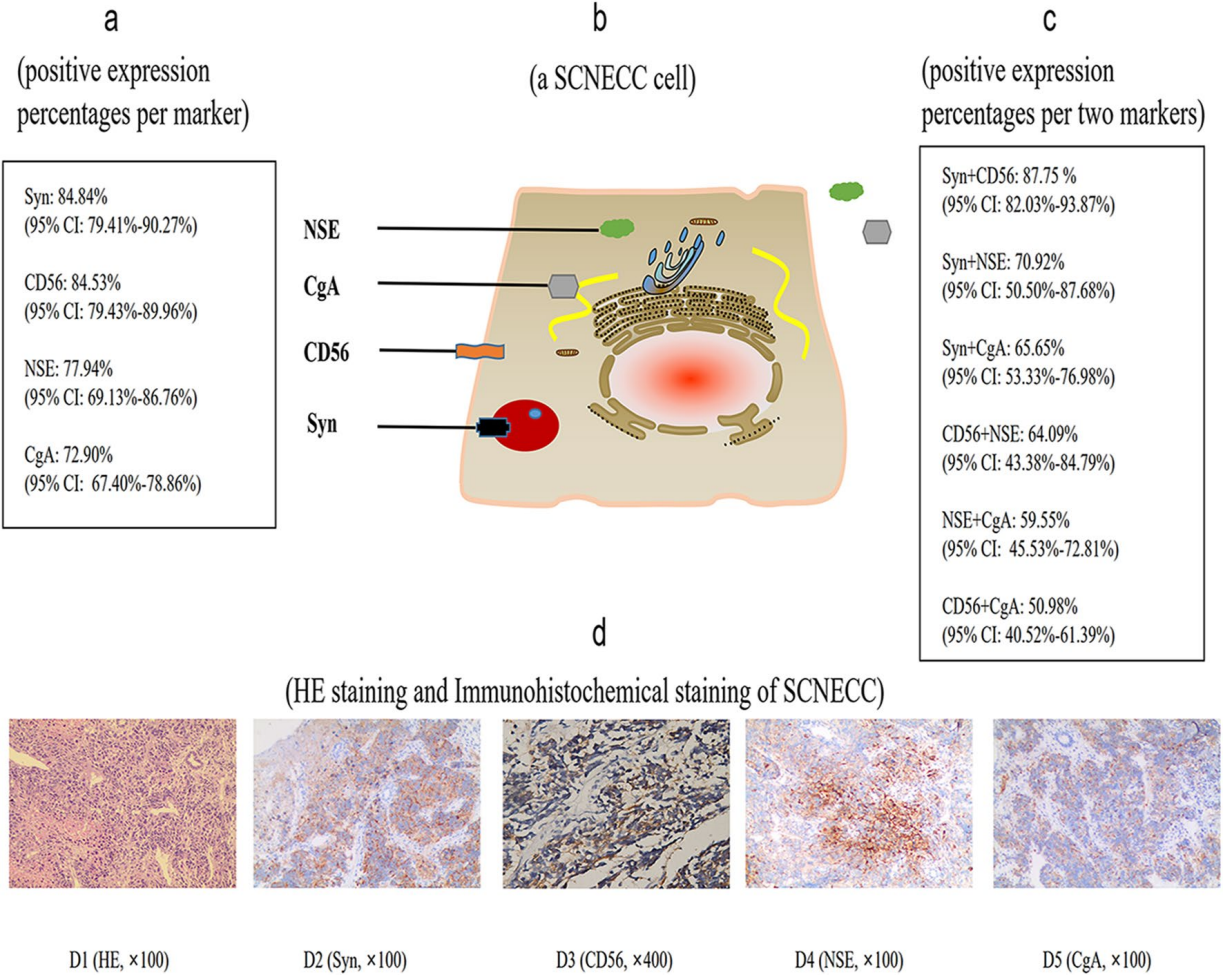
Brief overview of meta-analysis results. (**a**) (positive expression percentages per marker); (**b**) (a SCNECC cell), three proteins (containing Syn, CgA and NSE) locate in cell cytoplasm and only CD56 protein locates in the cell membrane; (**c**) (positive expression percentages per two markers); (**d**) (HE staining and Immunohistochemical staining of SCNECC), d1 (HE, × 100, d2 (Syn, × 100), d3 (CD56, × 400), d4 (NSE, × 100), d5 (CgA, × 100). All the pathological images come from our experiments. *Syn* synaptophysin; *CD56* neural cell adhesion molecules; *NSE* neuron-specific enolase; *CgA* chromograninA; *HE* hematoxylin–eosin; × 100, magnification 100 times; × 400, magnification 400 times.

Precise diagnosis is very crucial for SCNECC treatment. This disease requires specialized management recommendations depending on its unique biological behavior. Our results confirmed that the positive expression percentage of Syn was the highest among four classic neuroendocrine markers, and the positive rates of combination (Syn and CD56) were the highest among six combinations (Fig. 5). This is the first meta-analysis of the expression levels of neuroendocrine markers in SCNECC studies with the largest sample size. Therefore, the results of quantitative evaluation will help us select suitable markers for assisting diagnosing SCNECC.

The cancer cells of SCNECC have the similarity of neuroendocrine characteristics^44^. This is also the basis for distinguishing other morphologically similar tumors from SCNECC. Our study showed Syn had the highest expression rate, followed by CD56 in single marker expression. The combination Syn and CD56 have the highest positive expression rate in double marker expression simultaneously. The differences in the expression levels of four neuroendocrine markers are related to their molecular characteristics. Syn is a calcium binding protein located on the membrane of synapse vesicles, which diffusely expresses in the cytoplasm of neuroendocrine cells^4^. The molecular biological characteristic may explain why it is expressed with high degree. The expression level of CD56 is second only to Syn. And the heterogeneity of CD56 expression is smaller than those of other three markers. CD56 is a glycoprotein on the surface of cell membrane and also a member of cell adhesion molecule, which plays an important role in infiltration and metastasis of tumor cells^45^. The high expression level of CD56 corresponds with the aggressive properties of SCNECC. Moreover, CD56 has its own unique advantages in terms of stable expression detected by IHC method.

The positive expression rates of NSE and CgA markers are relatively low in our study, especially the expression level of CgA is the lowest. CgA and NSE are valuable markers for diagnosing neuroendocrine cancer, and their expressions are relevant to the patient's prognosis^6,12^.Our study did not reach a similar conclusion. The potential possibilities are as follows. Firstly, SCNECC may have a decline in the expression of some neuroendocrine cell characteristics for high degree of malignancy and poor differentiation. Secondly, expression rates of neuroendocrine markers may be affected by detection technology. The expressions of CgA and NSE can be detected by a serological assay, which were not included in our studies.

The 5-year overall survival rates of SCNECC range from 20 to 46.6%, and the prognosis of patients with advanced stage was very poor regardless of therapy^46^. However, SCNECC patients with early stage have the potential to receive multimodality therapy and have long term survival^47^. This difference emphasises the importance of early accurate diagnosis of SCNECC. There are some controversies in diagnostic criteria of SCNECC focusing on the necessity of neuroendocrine markers in the diagnosis. Some researchers pointed out that SCNECC was a morphologic diagnosis and the IHC evidence of neuroendocrine differentiation was not a requirement for diagnosis^25^. But actually, many studies have confirmed that accurate diagnosis of SNCECC require IHC staining of neuroendocrine markers which have been performed in clinical work too^1,3,12,19,22,29,48,49^. These findings highlighted the expression of two or more markers was a necessary criteria for diagnosing SCNECC.

The evidences reveal that differential diagnosis by neuroendocrine markers is particularly important in two situations. One is to differentiate SCNECC from other tumors with small cell morphological characteristics, and the other is to determine whether cervical adenocarcinoma or squamous carcinoma coexist with SCNECC^1,3,18^. With the development of diagnostic technology, it is found that SCNECC frequently occur mixed with other pathological types. Of the 42 studies enrolled in our study, 16 were patients with mixed SCNECC. To obtain more valuable results, we also analyzed the combined expression of two markers. We found that the combination (Syn and CD56) had the highest expression rate, which was consistent with the level of positive expression rate for single markers. Syn and CD56 are sensitive indicators for diagnosing SCNECC. However, the expression levels of both markers are highly variable.

Our study managed to collect almost all the related studies. However, the quantity, quality, and type of these studies still limited the level of evidence of this meta-analysis. All the included studies were retrospective types with small sample sizes. Heterogeneity of some studies existed in this meta-analysis. There were not adequate data and studies for the meta-analysis of prognosis. Since the data including IHC expressions of the four neuroendocrine markers in non-SCNECC were too small, it was not possible to compare the diagnostic specificity of these four markers. Thus, more studies including patients with non-small cell neuroendocrine cancer or clinical trials with a larger sample size are expected in the future.

## Conclusion

The positive expression percentage of Syn was the highest among four neuroendocrine markers, and the positive rates of combination (Syn and CD56) were the highest among six combinations. It is confirmed that Syn and CD56 are reliable indicators for diagnosing SCNECC.

## Supplementary information

Supplementary figure 1

Supplementary table 1

Supplementary table 2

Supplementary table 3

## Data Availability

All data generated and analysed during the study are included in this published article.
