# Supplementary material for: Diagnostic value of four neuroendocrine markers in small cell neuroendocrine carcinomas of the cervix: a meta-analysis
Source: Sci Rep. 2020 Sep 11;10:14975. doi: 10.1038/s41598-020-72055-x (PMC7486403; doi:10.1038/s41598-020-72055-x)
Supplement: Supplementary file 1 — Supplementary figure 1 [file 41598_2020_72055_MOESM1_ESM.pdf]

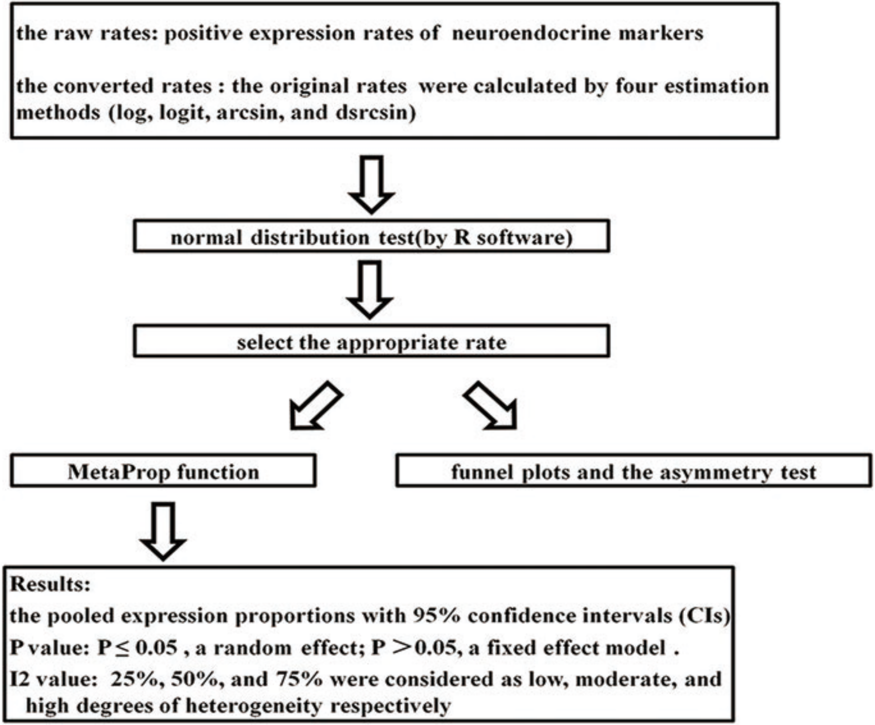
the raw rates: positive expression rates of neuroendocrine markers
the converted rates : the original rates were calculated by four estimation methods (log, logit, arcsin, and dsrcsin)
normal distribution test(by R software)
select the appropriate rate
MetaProp function
funnel plots and the asymmetry test
Results:
the pooled expression proportions with 95% confidence intervals (CIs)
P value: P ≤ 0.05 , a random effect; P >0.05, a fixed effect model .
I2 value: 25%, 50%, and 75% were considered as low, moderate, and high degrees of heterogeneity respectively
