# Supplementary material for: Diagnostic value of four neuroendocrine markers in small cell neuroendocrine carcinomas of the cervix: a meta-analysis
Source: Sci Rep. 2020 Sep 11;10:14975. doi: 10.1038/s41598-020-72055-x (PMC7486403; doi:10.1038/s41598-020-72055-x)
Supplement: Supplementary file 2 — Supplementary table 1 [file 41598_2020_72055_MOESM2_ESM.pdf]

Diagnostic value of four neuroendocrine markers in small cell neuroendocrine carcinomas of the cervix: a systematic review and meta-analysis

Rui Huang[1], Li Yu[1], Chunying Zheng[1], Qingchun Liang[2], Suye Suye[1], Xue Yang[1], Huan Yin[1], Zhen Ren[1], Liye Shi[1], Zhibang Zhang[1], Hongliang Chen[1], Chun Fu[1]

Supplementary Table 1. The sensitivity and specificity of neuroendocrine markers expression

Number	First author and year (Source of articles)	Study group number	Control group Number (Pathological type)	Sensitivity (%)	Specificity (%)
1	Ganesan 2016	23	18 (LCNECC)	CD56 (65%), Syn (83%), CgA (61%)	U
2	Kajiwara 2008	5	2 (LCNECC)	CD56 (80%), Syn (100%), CgA (80%)	U
3	Kuji 2017	29	8 (LCNECC)	CD56 (79%), Syn (86%), CgA (86%)	U
4	Inoue 1985	6	5 (ADC)	NSE (100%)	NSE (100%)
5	van 1988	15	18 (SCC)	CgA (20%), NSE (33%)	CgA (83%), NSE (83%)

6	Ueda 1989	10	4 (1ASC+3ADC)	CgA (40%), NSE (90%)	CgA (100%), NSE (71%)

Note: LCNECC, large cell neuroendocrine carcinoma of the cervix; ADCC, adenocarcinoma of the cervix; SCCC, squamous carcinoma of the cervix; ASCC, adenosquamous carcinoma of the cervix; U, unconsidered to be of clinical value because LCNECC also expresses neuroendocrine markers and the differentiation of the two tumors is based on cell morphology.
