# Supplementary material for: Diagnostic value of four neuroendocrine markers in small cell neuroendocrine carcinomas of the cervix: a meta-analysis
Source: Sci Rep. 2020 Sep 11;10:14975. doi: 10.1038/s41598-020-72055-x (PMC7486403; doi:10.1038/s41598-020-72055-x)
Supplement: Supplementary file 3 — Supplementary table 2 [file 41598_2020_72055_MOESM3_ESM.pdf]

Diagnostic value of four neuroendocrine markers in small cell neuroendocrine carcinomas of the cervix: a systematic review and meta-analysis

Rui Huang[1], Li Yu[1], Chunying Zheng[1], Qingchun Liang[2], Suye Suye[1], Xue Yang[1], Huan Yin[1], Zhen Ren[1], Liye Shi[1], Zhibang Zhang[1], Hongliang Chen[1], Chun Fu[1]

Supplementary Table 2. Quality assessment of included studies about case series

	1. Were there clear criteria for inclusion in the case series?	2. Was the condition measured in a standard, reliable way for all participants included in the case series?	3. Were valid methods used for identificati-on of the condition for all participant-s included in the case series?	4. Did the case series have consecutive inclusion of participants ?	5. Did the case series have complete inclusion of participants?	6. Was there clear reporting of the demographi cs of the participants in the study?	7. Was there clear reporting of clinical information of the participants?	8. Were the outcomes or follow up results of cases clearly reported?	9. Was there clear reporting of the presenting site(s)/clinic(s) demographic information?	10. Was statistical analysis appropriate?
Cheng 2008	Y	Y	Y	Y	U	Y	Y	Y	Y	U
Conner 2002	Y	Y	Y	Y	Y	Y	Y	Y	Y	U
Deng 2010	Y	Y	Y	Y	U	Y	Y	Y	Y	U
Emerson 2015	Y	Y	Y	Y	U	Y	Y	Y	Y	U
Ganesan 2016	Y	Y	Y	Y	N	Y	Y	Y	Y	Y

Giorgadze 2012	N	Y	Y	Y	N	Y	Y	Y	N	U
Horn 2006	Y	Y	Y	Y	Y	Y	Y	Y	Y	Y
Ishida 2004	Y	Y	Y	Y	U	Y	Y	Y	Y	U
Kajiwara 2008	Y	Y	Y	Y	U	Y	Y	Y	Y	Y
Kuji 2017	Y	Y	Y	Y	Y	Y	Y	Y	Y	Y
Li 2011	Y	Y	Y	Y	U	Y	Y	Y	Y	Y
Li 2013	Y	Y	Y	Y	U	Y	Y	N	N	U
Inoue 1985	U	Y	Y	Y	U	N	N	N	N	N
Qin 2011	Y	Y	Y	Y	U	Y	Y	Y	Y	Y
Rekhi 2012	Y	Y	Y	Y	U	Y	Y	Y	Y	Y
Sato 2003	Y	Y	Y	Y	U	Y	Y	Y	N	U
Sheridan 1996	Y	Y	Y	Y	U	Y	Y	Y	N	U
Sitthinamsuwan 2013	Y	Y	Y	Y	U	Y	Y	Y	Y	Y
Stoler 1991	Y	Y	Y	Y	Y	N	N	N	Y	Y
Straughn 2001	Y	Y	Y	Y	U	Y	Y	Y	Y	Y
Tsunoda 2005	Y	Y	Y	Y	U	Y	Y	Y	Y	U
Ueda 1989	Y	Y	Y	Y	U	N	N	N	N	U
Van 1988	Y	Y	Y	Y	U	Y	Y	Y	Y	Y
Viswanathan 2004	Y	Y	Y	Y	U	Y	Y	Y	Y	Y
Xing 2018	Y	Y	Y	Y	Y	Y	Y	Y	Y	U
Hu 2018	Y	Y	Y	Y	Y	Y	Y	Y	Y	Y
Han 2018	Y	Y	Y	Y	U	Y	Y	Y	Y	Y
Yang 2018	Y	Y	Y	Y	U	Y	Y	Y	Y	Y
Zeng 2018	Y	Y	Y	Y	U	Y	Y	Y	Y	U
Zhi 2018	Y	Y	Y	Y	U	Y	Y	Y	Y	Y

Wang 2019	Y	Y	Y	Y	U	Y	Y	Y	Y	Y
Tong 2018	Y	Y	Y	Y	U	Y	Y	Y	Y	Y
Wang 2018	Y	Y	Y	Y	U	Y	Y	N	Y	Y
Li 2015	Y	Y	Y	Y	U	Y	Y	Y	Y	Y
Morgan 2019	Y	Y	Y	Y	U	Y	Y	N	Y	Y
Liu 2018	Y	Y	Y	Y	U	Y	Y	N	Y	Y
Li 2018	Y	Y	Y	Y	U	N	N	N	N	Y
Jain 2019	Y	Y	Y	Y	U	Y	N	N	Y	Y
% positive response	95	100	100	100	16	89	87	76	82	63

Note: Y, yes; U, unknown; N no.
