# Supplementary material for: Diagnostic value of four neuroendocrine markers in small cell neuroendocrine carcinomas of the cervix: a meta-analysis
Source: Sci Rep. 2020 Sep 11;10:14975. doi: 10.1038/s41598-020-72055-x (PMC7486403; doi:10.1038/s41598-020-72055-x)
Supplement: Supplementary file 4 — Supplementary table 3 [file 41598_2020_72055_MOESM4_ESM.pdf]

Diagnostic value of four neuroendocrine markers in small cell neuroendocrine carcinomas of the cervix: a systematic review and meta-analysis

Rui Huang[1], Li Yu[1], Chunying Zheng[1], Qingchun Liang[2], Suye Suye[1], Xue Yang[1], Huan Yin[1], Zhen Ren[1], Liye Shi[1], Zhibang Zhang[1], Hongliang Chen[1], Chun Fu[1]

Supplementary Table 3. Quality assessment of one case report

	1.Were patient's demographic characteristics clearly described?	2.Was the patient's history clearly described and presented as a timeline?	3.Was the current clinical condition of the patient on presentation clearly described?	4.Were diagnostic tests or methods and the results clearly described?	5.Was the intervention(s) or treatment procedure(s) clearly described?	6.Was the post-intervention clinical condition clearly described?	7.Were adverse events (harms) or unanticipated events identified and described?	8.Does the case report provide takeaway lessons?
Fujii 1986	Y	Y	Y	Y	Y	Y	N	Y

Note: Y, yes; U, unknown; N no.
